# Persistent Fever and Positive PCR 90 Days Post-SARS-CoV-2 Infection in a Rituximab-Treated Patient: A Case of Late Antiviral Treatment

**DOI:** 10.3390/v14081757

**Published:** 2022-08-11

**Authors:** Nina Urke Ertesvåg, Sunniva Todnem Sakkestad, Fan Zhou, Ingrid Hoff, Trygve Kristiansen, Trygve Müller Jonassen, Elisabeth Follesø, Karl Albert Brokstad, Ruben Dyrhovden, Kristin G.-I. Mohn

**Affiliations:** 1 Influenza Centre, Department of Clinical Science, University of Bergen, 5021 Bergen, Norway; 2 Department of Medicine, Haukeland University Hospital, 5021 Bergen, Norway; 3 Department of Medical Biochemistry and Pharmacology, Haukeland University Hospital, 5021 Bergen, Norway; 4 Department of Thoracic Medicine, Haukeland University Hospital, 5021 Bergen, Norway; 5 Radiology Department, Haukeland University Hospital, 5021 Bergen, Norway; 6 Department of Safety, Chemistry and Biomedical Laboratory Sciences, Western Norway University of Applied Sciences, 5063 Bergen, Norway; 7 Department of Microbiology, Haukeland University Hospital, 5021 Bergen, Norway

**Keywords:** SARS-CoV-2, monoclonal antibodies, antivirals, human long-term infection, low-grade viral replication, B cell immune responses, hospitalized, persistent fever, immunocompromised, rituximab

## Abstract

**Background:** Persistent fever after SARS-CoV-2 infection in rituximab-treated patients has been reported. Due to reduced sensitivity in conventional sampling methods and unspecific symptoms in these patients, distinguishing between low-grade viral replication or hyperinflammation is challenging. Antiviral treatment is recommended as prophylactic or early treatment in the at-risk population; however, no defined treatment approaches for protracted SARS-CoV-2 infection exist. **Results:** We present a case of 96 days of persistent fever and SARS-CoV-2 infection in a patient receiving B cell depletion therapy for multiple sclerosis. Migratory lung infiltrates and positive PCR tests from serum (day-58 post infection) and lower airways (day-90 post infection) confirmed continuous viral replication. The dominant symptoms were continuous high fever, dyspnea and mild to moderate hypoxemia, which never developed into severe respiratory failure. The patient was hospitalized three times, with transient improvement after late antiviral treatment and full recovery 6 months post-rituximab infusion. **Conclusions:** A strategy for securing samples from lower airways and serum should be a prioritization to strengthen diagnostic certainty in immunocompromised patients. B-cell-deprived patients could benefit from late treatment with SARS-CoV-2-specific monoclonal antibodies and antivirals. Importantly, increased intervals between immunosuppressive therapy should be considered where feasible.

## 1. Introduction

As the SARS-CoV-2 pandemic continues to evolve, immunocompromised patients demonstrate protracted COVID-19 disease, ranging from mild clinical symptoms to severe infection [1,2,3,4]. Immunocompromised patients are a heterogenous group and may be either B or T cell deficient, or a combination of both, with varying ability to develop protective immune responses against SARS-CoV-2.

Rituximab is a monoclonal antibody (mAb), reactive to the pan B cell marker CD20, originally developed for B cell malignancies, but widely used to treat rheumatological, neurological and autoimmune diseases, including multiple sclerosis (MS) [5]. Rituximab clears B cells from blood and body tissues by inducing apoptosis, including most stages of B cell development and memory B cells [5]. Moreover, modulation of T cell responses may contribute to the anti-inflammatory effect of rituximab [6]. As a consequence, patients receiving rituximab have a reduced ability to elicit antibody responses after COVID-19 vaccination or SARS-CoV-2 infection, enabling prolonged, active viral replication once infected [7].

Neutralizing antibodies (elicited by vaccination or infection) inhibit infection and have been the main research focus during the COVID-19 pandemic. However, there is increasing knowledge concerning the central role of T cells in limiting severe disease [8]. Immunocompromised patients with a heightened risk of infection with novel SARS-CoV-2 may guide our understanding of specific immune cells’ interplay and mechanisms important to clear the infection. Passive immunity provided by mAb treatment may reduce progression to severe COVID-19 disease. However, there are no recommended antiviral treatments for persistent infections. Furthermore, the proper use of antiviral treatment in immunocompromised patients unable to control and eradicate a SARS-CoV-2 infection is uncertain. 

Here, we describe the protracted clinical course, diagnostic challenges and off-label antiviral treatment in a SARS-CoV-2-delta-variant-infected patient receiving rituximab. Full recovery from persistent fever and hypoxia required three hospital stays over a period of 102 days. The patient was treated with antivirals molnupiravir, remdesivir and the mAb sotrovimab, and was able to eradicate the infection when the rituximab effect faded. Although the patient never required intensive care treatment, she had severe symptoms and developed widespread lung tissue damage, resulting in long-term sick leave and reduced quality of life. 

## 2. Methods

### 2.1. Patient Consent and Ethical Approval

Written informed consent was obtained from the patient described in this case. The patient was enrolled as a part of a larger study approved by the Regional Ethics Committee (REK) Vest (#118664). 

### 2.2. In Vitro Viral Culture

A virus culture was set up in a certified Biosafety Level-3 facility in Bergen, Norway. In the first-round viral culture, patient respiratory samples from nasopharyngeal swabs were treated with gentamicin before inoculation with Vero cells in 6-well plates. After inoculation, Vero cells were cultured in DMEM with 1% FBS and antibiotics at 37 °C and were observed daily for cytopathic effect up to 6 days. In the second-round viral culture, supernatant from the first round Vero cell culture was inoculated with fresh Vero cells. After inoculation, Vero cells were cultured in DMEM with 1% FBS and antibiotics at 37 °C and were observed daily for cytopathic effect for another 6 days.

### 2.3. Microbiological Investigation

Samples from serum (S), naso/oropharyngeal swabs (NPS/OPS), bronchoalveolar lavage (BAL) and induced sputum (IS) were analyzed at the Department of Microbiology. SARS-CoV-2 RNA was analyzed on five different PCR platforms. If a cycle threshold (Ct) was required, RT-PCR analyses were performed with primers targeting the e gene of SARS-CoV-2 with either the automated Roche Flow System (Roche Molecular Systems, Laval, QC, Canada), Quant Studio-5 Real Time PCR System (Applied Biosystems^TM^, Waltham, MA, USA) or with the GeneXpert System (Cepheid, Sunnyvale, CA, USA). Other platforms used for SARS-CoV-2 RNA detection were the Roche Cobas Liat System (Roche Molecular systems, Laval, QC, Canada) and the TMA-based Panther System (Hologic, San Diego, CA, USA). 

To distinguish between the delta and the omicron SARS-CoV-2 variant the VirSNiP SARS-CoV-2 Spike 371L 373P 452R kit was used (TibMolBiol/Roche). The first two mutations indicate the omicron variant and the latter the delta variant. 

To detect the SARS-CoV-2 spike protein antigen (S-antigen), the CLIA-based LIAISON^®^ SARS-CoV-2 TrimericS IgG assay was used with the Liason^®^ XL (DIaSorin) instrument. The SARS-CoV-2 nucleocapsid antigen (N-antigen) was detected with SARS-CoV-2 IgG chemiluminescent microparticle immunoassay (CLIA) technology using the Alinity I System (Abbot).

## 3. Case Report

**Figure 1:**
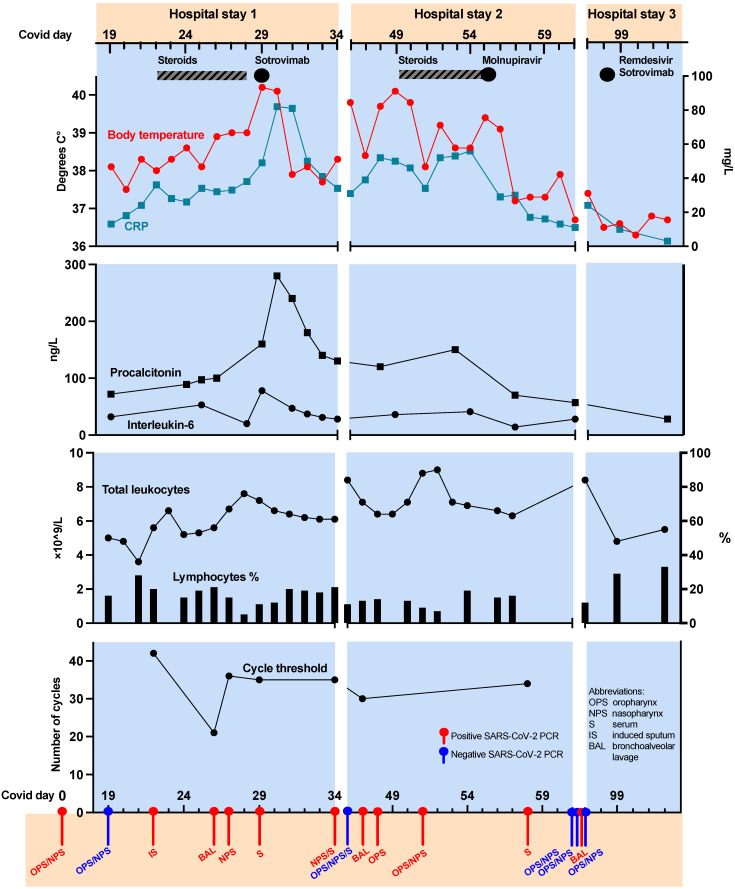
Overview of clinical course during three hospitalizations. Figure illustrates the clinical course of the COVID-19 infection, including body temperature measurements, selected biochemical parameters, PCR cycle threshold and SARS-CoV-2 PCR analyses over the course of three hospital stays, day 19–102. First hospital stay was days 19–34 post infection, the second hospitalization days 46–61 and third hospitalization days 97–102 post infection. In the top section, antiviral treatments administered are shown in black filled circles and steroids in black and grey striped segments. Level of C-reactive protein (CRP), procalcitonin and interleukin-6, lymphocyte percentage (%) are shown in the middle sections. The lower section includes PCR cycle threshold and SARS-CoV-2-positive samples in red and negative in blue. Abbreviations: OPS—oropharyngeal swab, NPS—nasopharyngeal swab, S—serum, IS—induced sputum, BAL—bronchoalveolar lavage.

**Figure 2:**
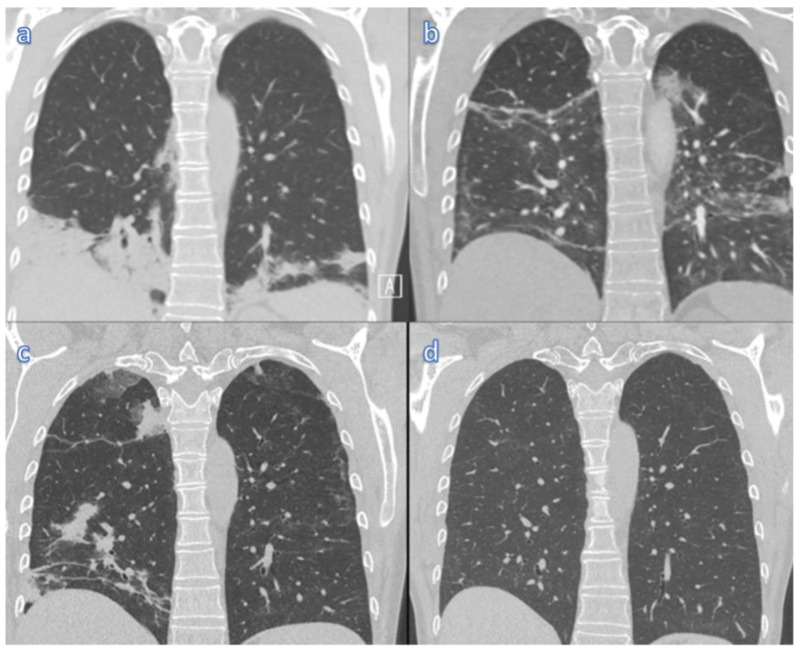
Coronal CT images of both lower lobes and caudal part of the upper lobes of the lungs. Coronal CT images of the lungs 21 (**a**), 46 (**b**), 82 (**c**) and 139 (**d**) days after initial infection. The first CT imaging 21 days after infection showed basal consolidations distributed peripherally and peribronchovascularly with air bronchograms. Mild bronchial dilation was surrounded by ground-glass opacities, most pronounced on the right side (**a**). At day 46 of disease, marked regression of right-sided changes were found, but new opacities had appeared on both sides (**b**). Similar changes with fluctuating opacities were seen day 82. The image illustrates right-sided regression and appearance of new opacities in the lower lobe and basal upper lobe (**c**). The latest follow-up at day 139 revealed almost complete resolution of the tissue changes, with only a few subtle remaining ground-glass opacities (**d**). Imprints like these may persist for several months post infection.

A 59-year-old, non-smoking, full-time-working engineer with a history of cured breast cancer and rituximab-treated relapsing-remitting multiple sclerosis (RRMS) was admitted due to persistent fever 9 days after testing positive for SARS-CoV-2, in January 2022 (Figure 1). On admission, vital parameters were within normal range (BP 121/83 mmHg, HR 83/min, temperature 37.3 °C, respiratory frequency 16/min, SpO2 100%). Lung auscultation revealed fine crackles bilaterally. Laboratory analysis showed a slightly elevated CRP (13 mg/L) and Interleukin-6 (32 ng/L), as well as a reduced lymphocyte count (0.8 × 10^9^/L) and a slightly elevated D-dimer (1.05 mg/L), otherwise blood tests were within normal range. A CT scan confirmed pneumonia and excluded pulmonary embolism (Table 1, Figure 2a). Initial RT-PCR testing (OPS/NPS) was negative for SARS-CoV-2, but positive in induced sputum (IS) a few days later, confirming the delta variant (Ct value of 42). She had received the novel mRNA vaccines while under treatment with rituximab (500 mg infusions every 6 months). The first vaccination (Corminarty) occurred one month after rituximab treatment and subsequent booster doses (Corminarty, SpikeVax) were administered within 5 months, followed by a new rituximab infusion two months later. Consequently, no SARS-CoV-2 IgG was detected at admission, including anti-spike (S) and anti-nucleocapsid (N) antibodies.

The first days in hospital, the patient’s condition worsened with significant speech dyspnea and febrile episodes twice daily (up to 40.2 °C), SpO2 was 95% at rest and 71–83% during light activities and oxygen supplementation was commenced. Prednisolone 20–40 mg daily was administered days 22–28 post infection, for suspected COVID-19 hyperinflammation, but to no effect. Bronchoscopy was macroscopically normal; however, BAL specimen was SARS-CoV-2 PCR positive, with a lower Ct value (Ct 21) compared to the upper airways (Ct 35), consistent with COVID-19 pneumonia (Supplementary Table S1). A Vero cell culture was set up to attempt propagation of SARS-CoV-2 virus from the NPS sample taken on day 27. A course of cefotaxim did not improve her symptoms. Sotrovimab 500 mg iv was administered day 29 against suspected low-grade viremia resulting in rapid general improvement and less dyspnea, and she was discharged.

The patient was readmitted on day 46 with a relapse of high fever (40.0 °C), chills and speech dyspnea, but negative SARS-CoV-2 PCR from both OPS/NPS and serum. SARS-CoV-2 IgG antibodies against the S-antigen, but not the N-antigen, were detected for the first time, suspected due to the recent sotrovimab treatment and not an endogenous response to the infection. A repeat CT scan revealed both regression and progression, with new consolidations and ground-glass opacities bilaterally (Figure 2b). Extended CT abdominal and pelvic scan ruled out malignancies and other pathology. BAL was repeated to differentiate between persistent COVID-19 infection, secondary infections and interstitial lung disease. SARS-CoV-2 RNA was, again, positive, with a higher Ct value (Ct 30) compared to sampling 3 weeks prior (Ct 21). No other microbiological agents were detected, including *Pneumocystis jirovecii.* Blood screening for other infectious diseases and auto-antibodies was negative (Supplementary Table S1). 

A second high-dose prednisolone treatment course (50 mg/day for 6 days) was initiated on day 49 without effect. Pulmonary function tests revealed significantly impaired lung function, compared to completely normal results in November 2021 (spirometry, diffusing capacity of the lungs for carbon monoxide (DLCO), body box) (Table 2). 

She had persistent fever and resting hypoxemia (SpO2 91–93%). The SARS-CoV-2 omicron variant dominated in Norway at this time; however, a repeat PCR reconfirmed the delta variant. Hence, available antiviral treatment (molnupiravir) was administered on day 55 with a rapid response, with receding fever, dyspnea and CRP (Figure 1). An NPS PCR was negative for SARS-CoV-2 RNA and she was discharged for the second time on day 61. 

A follow-up outpatient CT scan on day 82 revealed peripheral ground-glass opacities and consolidations, with varied improvement and progression (Figure 2c). She reported renewed low-grade fever and dyspnea. A third bronchoscopy again confirmed SARS-CoV-2 delta variant (Ct 33) and she was readmitted on day 97 for a combined 5-day antiviral treatment (remdesivir) and sotrovimab infusion. She was discharged five days later feeling well and fever free. A completely normalized spirometry and nearly full resolution of the lung-CT pathology was confirmed at the outpatient clinic 179 days after infection (Figure 2d, Table 2). 

## 4. Discussion

### 4.1. Prolonged SARS-CoV-2 Infection and Immunological Aspects

More than two years into the COVID-19 pandemic, protracted or more severe disease following infection with SARS-CoV-2 among immunocompromised patients has been reported [9,10,11,12,13]. We present a B-cell-depleted patient, suffering from persistent fever, hypoxemia, but not severe disease, 96 days post-SARS-CoV-2 infection. She received three COVID-19 vaccinations, but never mounted measurable SARS-CoV-2 IgG antibody responses. The long reconstitution period of the B-cell population after rituximab treatment (6 to 12 months) [14,15], and thereby, a poor autologous B-cell function, probably contributed to her long-drawn COVID-19 disease.

Interestingly, her persistent low-grade symptoms never developed into severe disease, reflecting perhaps a functional T-cell component that had not been significantly inhibited by rituximab. Cellular immunity provides protection from severe disease [8] and likely supported infection control by killing virus-infected cells, but was not sufficient to clear the virus. Paradoxically, her immunocompromised state may also have protected her from developing severe disease by limiting COVID-19 hyperinflammatory syndrome characterized by massive cytokine secretion [16]. While rapid and significant biochemical and clinical improvement occurred during antiviral treatments, we believe these therapies augmented and worked in concert with her own immune response to eradicate the virus. Full recovery took place 7 months after her last rituximab infusion with improved autologous B-cell function. 

### 4.2. Diagnostic Considerations

This case demonstrates the challenge in diagnosing low-grade SARS-CoV-2 viral infection in an immunocompromised patient. Repeated CT scans of the lungs revealed migratory opacities consistent with a pattern of organizing pneumonia (OP). After excluding other possible causes, our two main differential diagnoses were persistent low-grade SARS-CoV-2 viral replication or COVID-19-induced hyperinflammation, resulting in widespread lung tissue damage (Figure 2). OP is classified as either cryptogenic (COP) or secondary (SOP). Our patient was considered to have SOP, as a radiologic pattern of OP is common in COVID-19 [17]. Corticosteroids are the standard treatment for OP and clinical improvement normally occurs within 2–3 days [18]. Our patient showed no improvement, despite high doses of steroids. This led to the suspicion of ongoing SARS-CoV-2 infection as the cause of her persistent fever and hypoxemia. 

Securing multiple and representative patient samples is important for the differential diagnosis of other infectious agents. PCR testing of BAL or serum has been recommended as the optimal diagnostic tool in rituximab treated patients with COVID-19, based on reduced sensitivity of NPS and OPS [10]. While SARS-CoV-2 RNA was consistently detected in all BAL samples and in three serum samples, sampling from the upper airways varied between being RT-PCR negative or positive, with a high Ct value (Figure 1), making interpretation difficult. A sample with a high Ct value can be synonymous with poor sampling technique, but in the presented case, we believe more likely attributed to a lower amount of virus in the upper airways [19]. 

Another consideration was if the detection of viral RNA represented active viral replication or dead virus from a cleared infection. In our attempt to propagate SARS-CoV-2 virus in Vero cell culture, a mild cytopathic effect (CPE) was observed initially; however, no CPE was seen in the second passage. A potential explanation could be that the virus had acquired receptor-binding mutations after a prolonged infection period in the host. The patient received intermittent courses of antiviral treatment that could have facilitated events of viral escape. Consequently, the Vero cells were no longer efficient host cells for virus replication and the viruses were lost in the in vitro culture, a phenomenon reported for influenza A/H3N2-viruses and SARS-CoV-2 omicron [20,21].

Our hypothesis of active viral replication is supported by the longevity of SARS-CoV-2-positive BAL (day 90) and serum (day 58) samples (Figure 1). A meta-analysis, investigating the longevity of SARS-CoV-2 viral detection in lower airways, found a mean and max duration of 14.6 and 59 days, respectively [22]. To the best of our knowledge, no previous studies have reported 90 days viral shedding in the lower airways [22]. However, few studies have focused on the duration of viral shedding in immunocompromised patients. Studies on the duration of SARS-CoV-2 RNA detection in serum of hospitalized patients found a markedly shorter detection period (15 and 16 days) than in our presented case (58 days) [23,24]. The detection of viral RNA in serum is associated with a poorer patient outcome and increased disease severity and, most importantly, a sign of active viral replication [10,23,24,25,26]. However, these findings are based on few studies and viral presence in serum could differ among SARS-CoV-2 variants.

### 4.3. Antiviral Treatments

SARS-CoV-2-specific mAbs and antiviral treatments are approved for prophylactic or early post-exposure treatment of patients with risk of severe COVID-19 disease, especially in immunocompromised patients with a poor host response. Antivirals are generally well tolerated, but a limited, expensive resource. Our patient was not within the recommended timeframe for treatment, but without other treatment options, three specific antiviral drugs were administered (Figure 1). 

Sotrovimab is a pan-sarbecovirus mAb developed for treatment of COVID-19. The mAb binds and neutralizes the most common epitopes on the spike protein, including the delta variant. It is primarily intended for early treatment in non-hospitalized high-risk patients, since no clinical improvement was found amongst hospitalized patients with COVID-19 [27,28]. Remdesivir, a nucleoside analog, is not recommended for patients who do not require supplemental oxygen, but may be given to hospitalized patients with a high risk of severe disease progression [29]. A transient improvement of symptoms occurred after administration of all antiviral treatments. Specifically, after three doses of molnupiravir, a ribonucleoside analog shown to reduce risk of hospitalization and death [30,31] and the fever subsided for a week. However, the fever relapsed and our patient did not experience full recovery until a second course of sotrovimab in combination with remdesivir, administered on day-97 post infection. 

Increasing immune pressure in the population caused by vaccination and previous infections drives the rapid mutational rate of SARS-CoV-2, challenging current mAbs’ sensitivity. This has resulted in the mAbs developed towards earlier variants now being significantly less effective against the circulating omicron variants (BA.2.12.1, BA.4/BA.5) [32]. Similarly, frequent use of antivirals increases the risk of drug resistance. Hence, vaccines are preferred compared to post-exposure treatments. However, effective antiviral treatments, perhaps in combination, are required for patients unable to respond to conventional vaccines. There is a need for defined treatment approaches and careful clinical strategies regarding the use of antivirals to reduce the risk of viral adaptation and resistance in this patient group. 

## 5. Conclusions

Limited knowledge of immunological mechanisms prolonging acute SARS-CoV-2 infections in patients with profound immune dysregulation challenge clinical diagnostics and treatment options. This case demonstrates the complex dynamic between infection and disease in a small, but important, group of patients unable to eradicate the virus.

Careful patient-customized antiviral therapy may have a role beyond the initial days of infection in patients with protracted COVID-19 disease. However, B cells may be essential in clearing SARS-CoV-2 infections in rituximab-treated patients; hence, longer intervals between infusions should be considered. Additionally, to aid the certainty of low-grade COVID-19 viral replication, SARS-CoV-2 PCR analysis of BAL and serum is recommended.

## Figures and Tables

**Table 1:** Development in COVID-19 relevant biochemical parameters during first hospital stay.

Analysis	Unit	Week of First Hospital Stay			Reference Interval
		1	2	3	
Hemoglobin	g/dL	11.8	11.4	10.5	11.7–15.3
Total leukocytes	×10^9^/L	5.0	5.3	6.1	4.1–9.8
Neutrophils	×10^9^/L	3.7	3.7	3.9	1.8–6.9
Lymphocytes	×10^9^/L	0.8	1.0	1.3	1.2–3.1
Monocytes	×10^9^/L	0.49	0.57	0.83	0.28–0.90
Eosinophils	×10^9^/L	0.0	0.0	0.1	≤0.5
Basophils	×10^9^/L	0.01	0.02	0.03	≤0.10
Thrombocytes	×10^9^/L	224	346	591	165–387
CRP	mg/L	13	34	34	<5
Interleukin-6	ng/L	32	53	28	0–7
Procalcitonin	µg/L	<0.10	<0.10	0.13	<0.10
Ferritin	µg/L	65	92	215	18–240
PT-INR	₋	0.9	1.0	0.9	0.9–1.2
APTT	s	24	26	₋	22–30
Fibrinogen	g/L	4.8	5.1	₋	1.9–4.0
D-dimer	mg/L FEU	1.05	1.3	₋	<0.27
ALAT	U/L	17	86	303	10–45
ASAT	U/L	35	71	227	15–35
ALP	U/L	82	80	161	35–105
LD	U/L	228	195	295	105–205

Abbreviations: CRP—C-Reactive Protein, PT-INR—Prothrombin-International Normalized Ratio, APTT—Activated Partial Thromboplastin Time, ALAT—Alanine Aminotransferase ASAT—Aspertate Aminotransferase, ALD—alkaline phosphatase, LD—lactate dehydrogenase, FEU—fibrinogen equivalent units.

**Table 2:** Spirometry values before, during and after COVID-19 disease.

COVID Day	FVC (L)	FVC (% of Predicted)	FEV1 (L)	FEV1/FVC (%)	DLCO (mmol/min × kPa)	TLC (L)	TLC (% of Predicted)
Pre-illness	3.8	114	3.0	78	8.0	5.4	104
50	2.1	61	1.8	87	2.6	2.8	54
89	2.3	68	1.9	82	3.0	3.1	60
109	3.6	106	2.8	79	4.0	4.2	81
179	3.1	93	2.6	85	7.2	5.4	111

Abbreviations: FVC—forced vital capacity, FEV1—forced expiratory volume in the 1st s, DLCO—diffusion capacity for carbon monoxide, TLC—total lung capacity.

## Data Availability

Not applicable.

## Supplementary Materials

The following supporting information can be downloaded at: https://www.mdpi.com/article/10.3390/v14081757/s1, Table S1: Supplementary microbiological and immunological analyses title.

## References

[1] Gaitzsch E., Passerini V., Khatamzas E., Strobl C.D., Muenchhoff M., Scherer C., Osterman A., Heide M., Reischer A., Subklewe M. (2021). COVID-19 in Patients Receiving CD20-depleting Immunochemotherapy for B-cell Lymphoma. Hemasphere.

[2] Choudhary M.C., Crain C.R., Qiu X., Hanage W., Li J.Z. (2021). Severe Acute Respiratory Syndrome Coronavirus 2 (SARS-CoV-2) Sequence Characteristics of Coronavirus Disease 2019 (COVID-19) Persistence and Reinfection. Clin. Infect. Dis..

[3] Choi B., Choudhary M.C., Regan J., Sparks J.A., Padera R.F., Qiu X., Solomon I.H., Kuo H.H., Boucau J., Bowman K. (2020). Persistence and Evolution of SARS-CoV-2 in an Immunocompromised Host. N. Engl. J. Med..

[4] Baang J.H., Smith C., Mirabelli C., Valesano A.L., Manthei D.M., Bachman M.A., Wobus C.E., Adams M., Washer L., Martin E.T. (2021). Prolonged Severe Acute Respiratory Syndrome Coronavirus 2 Replication in an Immunocompromised Patient. J. Infect. Dis..

[5] Weiner G.J. (2010). Rituximab: Mechanism of action. Semin. Hematol..

[6] Chisari C.G., Sgarlata E., Arena S., Toscano S., Luca M., Patti F. (2022). Rituximab for the treatment of multiple sclerosis: A review. J. Neurol..

[7] Iyer R.B., Raghavendra S., Nooraine J., Jaychandran R. (2022). COVID-19 outcomes in persons with multiple sclerosis treated with rituximab. Mult. Scler. Relat. Disord..

[8] Moss P. (2022). The T cell immune response against SARS-CoV-2. Nat. Immunol..

[9] Rabascall C.X., Lou B.X., Navetta-Modrov B., Hahn S.S. (2021). Effective use of monoclonal antibodies for treatment of persistent COVID-19 infection in a patient on rituximab. BMJ Case Rep..

[10] Furlan A., Forner G., Cipriani L., Vian E., Rigoli R., Gherlinzoni F., Scotton P. (2021). COVID-19 in B Cell-Depleted Patients After Rituximab: A Diagnostic and Therapeutic Challenge. Front. Immunol..

[11] Baker D., Roberts C.A.K., Pryce G., Kang A.S., Marta M., Reyes S., Schmierer K., Giovannoni G., Amor S. (2020). COVID-19 vaccine-readiness for anti-CD20-depleting therapy in autoimmune diseases. Clin. Exp. Immunol..

[12] Burgener S., Rochat P., Dollenmaier G., Benz G., Kistler A.D., Fulchini R. (2022). Progression of COVID-19 in a Patient on Anti-CD20 Antibody Treatment: Case Report and Literature Review. Case Rep. Infect. Dis..

[13] Kos I., Balensiefer B., Roth S., Ahlgrimm M., Sester M., Schmidt T., Thurner L., Bewarder M., Bals R., Lammert F. (2020). Prolonged Course of COVID-19-Associated Pneumonia in a B-Cell Depleted Patient After Rituximab. Front. Oncol..

[14] Ng C.M., Bruno R., Combs D., Davies B. (2005). Population pharmacokinetics of rituximab (anti-CD20 monoclonal antibody) in rheumatoid arthritis patients during a phase II clinical trial. J. Clin. Pharmacol..

[15] Hogan J., Dossier C., Kwon T., Macher M.A., Maisin A., Couderc A., Niel O., Baudouin V., Deschênes G. (2019). Effect of different rituximab regimens on B cell depletion and time to relapse in children with steroid-dependent nephrotic syndrome. Pediatr. Nephrol..

[16] Fung M., Babik J.M. (2021). COVID-19 in Immunocompromised Hosts: What We Know So Far. Clin. Infect. Dis..

[17] Wang Y., Jin C., Wu C.C., Zhao H., Liang T., Liu Z., Jian Z., Li R., Wang Z., Li F. (2020). Organizing pneumonia of COVID-19: Time-dependent evolution and outcome in CT findings. PLoS ONE.

[18] de Oliveira Filho C.M., Vieceli T., de Fraga Bassotto C., da Rosa Barbato J.P., Garcia T.S., Scheffel R.S. (2021). Organizing pneumonia: A late phase complication of COVID-19 responding dramatically to corticosteroids. Braz. J. Infect. Dis..

[19] Dahdouh E., Lázaro-Perona F., Romero-Gómez M.P., Mingorance J., García-Rodriguez J. (2021). C(t) values from SARS-CoV-2 diagnostic PCR assays should not be used as direct estimates of viral load. J. Infect..

[20] Allen J.D., Ross T.M. (2018). H3N2 influenza viruses in humans: Viral mechanisms, evolution, and evaluation. Hum. Vaccin. Immunother..

[21] Mautner L., Hoyos M., Dangel A., Berger C., Ehrhardt A., Baiker A. (2022). Replication kinetics and infectivity of SARS-CoV-2 variants of concern in common cell culture models. Virol. J..

[22] Cevik M., Tate M., Lloyd O., Maraolo A.E., Schafers J., Ho A. (2021). SARS-CoV-2, SARS-CoV, and MERS-CoV viral load dynamics, duration of viral shedding, and infectiousness: A systematic review and meta-analysis. Lancet Microbe..

[23] Hagman K., Hedenstierna M., Rudling J., Gille-Johnson P., Hammas B., Grabbe M., Jakobsson J., Dillner J., Ursing J. (2022). Duration of SARS-CoV-2 viremia and its correlation to mortality and inflammatory parameters in patients hospitalized for COVID-19: A cohort study. Diagn. Microbiol. Infect. Dis..

[24] Zheng S., Fan J., Yu F., Feng B., Lou B., Zou Q., Xie G., Lin S., Wang R., Yang X. (2020). Viral load dynamics and disease severity in patients infected with SARS-CoV-2 in Zhejiang province, China, January-March 2020: Retrospective cohort study. BMJ.

[25] Jacobs J.L., Mellors J.W. (2020). Detection of Severe Acute Respiratory Syndrome Coronavirus 2 (SARS-CoV-2) RNA in Blood of Patients With Coronavirus Disease 2019 (COVID-19): What Does It Mean?. Clin. Infect. Dis..

[26] Bermejo-Martin J.F., González-Rivera M., Almansa R., Micheloud D., Tedim A.P., Domínguez-Gil M., Resino S., Martín-Fernández M., Ryan Murua P., Pérez-García F. (2020). Viral RNA load in plasma is associated with critical illness and a dysregulated host response in COVID-19. Crit. Care.

[27] Gupta A., Gonzalez-Rojas Y., Juarez E., Crespo Casal M., Moya J., Falci D.R., Sarkis E., Solis J., Zheng H., Scott N. (2021). Early Treatment for COVID-19 with SARS-CoV-2 Neutralizing Antibody Sotrovimab. N. Engl. J. Med..

[28] Self W.H., Sandkovsky U., Reilly C.S., Vock D.M., Gottlieb R.L., Mack M., Golden K., Dishner E., Vekstein A., Ko E.R. (2022). Efficacy and safety of two neutralising monoclonal antibody therapies, sotrovimab and BRII-196 plus BRII-198, for adults hospitalised with COVID-19 (TICO): A randomised controlled trial. Lancet Infect. Dis..

[29] The National Institutes of Health Therapeutic Management of Hospitalized Adults with COVID-19. https://www.covid19treatmentguidelines.nih.gov/management/clinical-management/hospitalized-adults--therapeutic-management/.

[30] Jayk Bernal A., Gomes da Silva M.M., Musungaie D.B., Kovalchuk E., Gonzalez A., Delos Reyes V., Martín-Quirós A., Caraco Y., Williams-Diaz A., Brown M.L. (2022). Molnupiravir for Oral Treatment of COVID-19 in Nonhospitalized Patients. N. Engl. J. Med..

[31] Singh A.K., Singh A., Singh R., Misra A. (2021). Molnupiravir in COVID-19: A systematic review of literature. Diabetes Metab. Syndr..

[32] Arora P., Kempf A., Nehlmeier I., Schulz S.R., Cossmann A., Stankov M.V., Jäck H.-M., Behrens G.M.N., Pöhlmann S., Hoffmann M. (2022). Augmented neutralisation resistance of emerging omicron subvariants BA.2.12.1, BA.4, and BA.5. Lancet Infect. Dis..

