# Supplementary material for: Persistent Fever and Positive PCR 90 Days Post-SARS-CoV-2 Infection in a Rituximab-Treated Patient: A Case of Late Antiviral Treatment
Source: Viruses. 2022 Aug 11;14(8):1757. doi: 10.3390/v14081757 (PMC9414720; doi:10.3390/v14081757)
Supplement: Supplementary file 1 [file viruses-14-01757-s001.zip › viruses-1830537-supplementary.pdf]

Supplementary Table S1: Supplementary laboratory analyses performed to exclude other fever-causing conditions

Material	Analysis	Result
Blood	Cultivation	Negative
	Infectious diseases serology:	
	Brucella (IgM, IgG, IgA)	Negative
	Coxiella (IgM, IgG)	Negative
	Epstein-Barr virus EBNA IgG	Positive*
	Epstein-Barr virus DNA	Negative
	Borrelia burgdorferi (IgM, IgG)	Negative
	Toxoplasma gondii (IgM, IgG)	Negative
	Francisella tularensis antibodies	Negative
	Cytomegalovirus (IgM, IgG)	Negative
	Cytomegalovirus DNA	Negative
	Hepatitis A	Immunoprotection*
	Hepatitis B	Immunoprotection*
	Hepatitis C	Negative
	HIV antigen/antibodies	Negative
	Autoimmune disease screening:	
	Rheumatoid factor	Negative
	Anti-CCP (IgG)	Negative
	Anti-myeloperoxidase antibodies	Negative
	Anti-proteinase3 antibodies	Negative
	Anti-actin IgG	Negative
	Anti-mitochondria antibodies	Negative
	Anti-LKM1 IgG	Negative
	Anti-ANA IgG	Positive*
	Anti-Sm IgG	Negative
	Anti-RNP IgG	Negative
	Anti-SSA IgG	Negative
	Anti-SSB IgG	Negative
	Anti-Jo1 IgG	Negative
	Anti-Scl-70 IgG	Negative
	Anti-centromer IgG	Positive*
	Anti-dsDNA IgG	Negative
	Anti-chromatin antibodies	Negative
	Anti-ribosomal P IgG	Negative
	Anti-Sm RNP IgG	Negative
	Neuroendocrine disease screening:	
	Metanefrine	Negative
	Normetanefrine	Negative
	Metoksytyramine	Negative
Bronchoalveolar lavage	Cultivation bacteria and fungi	Negative
	Cytology	No atypical cells
	Respiratory pathogens PCR:	
	Aspergillus species	Negative
	Mucor	Negative
	Bordatella pertussis	Negative
	Bordatella parapertussis	Negative
	Mycoplasma pneumoniae	Negative
	Chlamydia pneumoniae	Negative
	Legionella species	Negative

	Pneumocystis jirovecii<br>Influensavirus A and B<br>Parainfluensavirus 1, 2 and 3<br>Respiratory syncytialvirus<br>Human metapneumovirus<br>Rhinovirus<br>SARS-CoV-2	Negative<br>Negative<br>Negative<br>Negative<br>Negative<br>Negative<br>Positive*
Oropharynx/ nasopharynx	Respiratory pathogens PCR:<br>*Bordatella pertussis*<br>*Bordatella parapertussis*<br>*Mycoplasma pneumoniae*<br>*Chlamydia pneumoniae*<br>Influensavirus A and B<br>Parainfluensavirus 1, 2 and 3<br>Respiratory syncytialvirus<br>Human metapneumovirus<br>Rhinovirus<br>SARS-CoV-2	 Negative<br>Negative<br>Negative<br>Negative<br>Negative<br>Negative<br>Negative<br>Negative<br>Negative<br>Positive*
Urine	Cultivation<br>*Pneumococci* antigen<br>*Legionella* antigen	Negative<br>Negative<br>Negative
Feces	Enteric pathogens PCR	Negative
